# Class A Penicillin-Binding Protein C Is Responsible for Stress Response by Regulation of Peptidoglycan Assembly in Clavibacter michiganensis

**DOI:** 10.1128/spectrum.01816-22

**Published:** 2022-08-30

**Authors:** Xing Chen, Yao Li, Kaihong Bai, Meng Gu, Xiaoli Xu, Na Jiang, Yu Chen, Jianqiang Li, Laixin Luo

**Affiliations:** a Department of Plant Pathology and MOA Key Laboratory of Pest Monitoring and Green Management, College of Plant Protection, Beijing Key Laboratory of Seed Disease Testing and Control, China Agricultural University, Beijing, People’s Republic of China; b Key Laboratory of Integrated Crop Pest Management of Anhui Province, Key Laboratory of Biology and Sustainable Management of Plant Diseases and Pests of Anhui Higher Education Institutes, School of Plant Protection, Anhui Agricultural University, Hefei, People’s Republic of China; University of Guelph

**Keywords:** PBPC, peptidoglycan, thickness, crosslinkage, stress response, cell viability, cell wall

## Abstract

The cell wall peptidoglycan of bacteria is essential for their survival and shape development. The penicillin-binding proteins (PBPs) are responsible for the terminal stage of peptidoglycan assembly. It has been shown that PBPC, a member of class A high-molecular-weight PBP, played an important role in morphology maintenance and stress response in Clavibacter michiganensis. Here, we reported the stress response strategies under viable but nonculturable (VBNC) state and revealed the regulation of peptidoglycan assembly by PBPC in C. michiganensis cells. Using atomic force microscopy imaging, we found that peptidoglycan of C. michiganensis cells displayed a relatively smooth and dense surface, whereas *ΔpbpC* was characterized by a “ridge-and-groove” surface, which was more distinctive after Cu^2+^ treatment. The peptidoglycan layer of wild type cells exhibited a significant increase in thickness and slight increase in cross-linkage following Cu^2+^ treatment. Compared with wild type, the thickness and cross-linkage of peptidoglycan decreased during log phase in *ΔpbpC* cells, but the peptidoglycan cross-linkage increased significantly under Cu^2+^ stress, while the thickness did not change. It is noteworthy that the above changes in the peptidoglycan layer resulted in a significant increase in the accumulation of amylase and exopolysaccharide in *ΔpbpC*. This study elucidates the role of PBPC in Gram-positive rod-shaped plant pathogenic bacterium in response to environmental stimuli by regulating the assembling of cell wall peptidoglycan, which is significant in understanding the survival of C. michiganensis under stress and the field epidemiology of tomato bacterial canker disease.

**IMPORTANCE** Peptidoglycan of cell walls in bacteria is a cross-linked and meshlike scaffold that provides strength to withstand the external pressure. The increased cross-linkage in peptidoglycan and altered structure in VBNC cells endowed the cell wall more resistant to adversities. Here we systematically evaluated the stress response strategies in Gram-positive rod-shaped bacterium C. michiganensis log phase cells and revealed a significant increase of peptidoglycan thickness and slight increase of cross-linkage after Cu^2+^ treatment. Most strikingly, knocking-out of PBPC leads to a significant increase in cross-linking of peptidoglycan in response to Cu^2+^ treatment. Understanding the stress resistance mechanism and survival strategy of phytopathogenic bacteria is the basis of exploring bacterial physiology and disease epidemiology.

## INTRODUCTION

**FIG 1:**
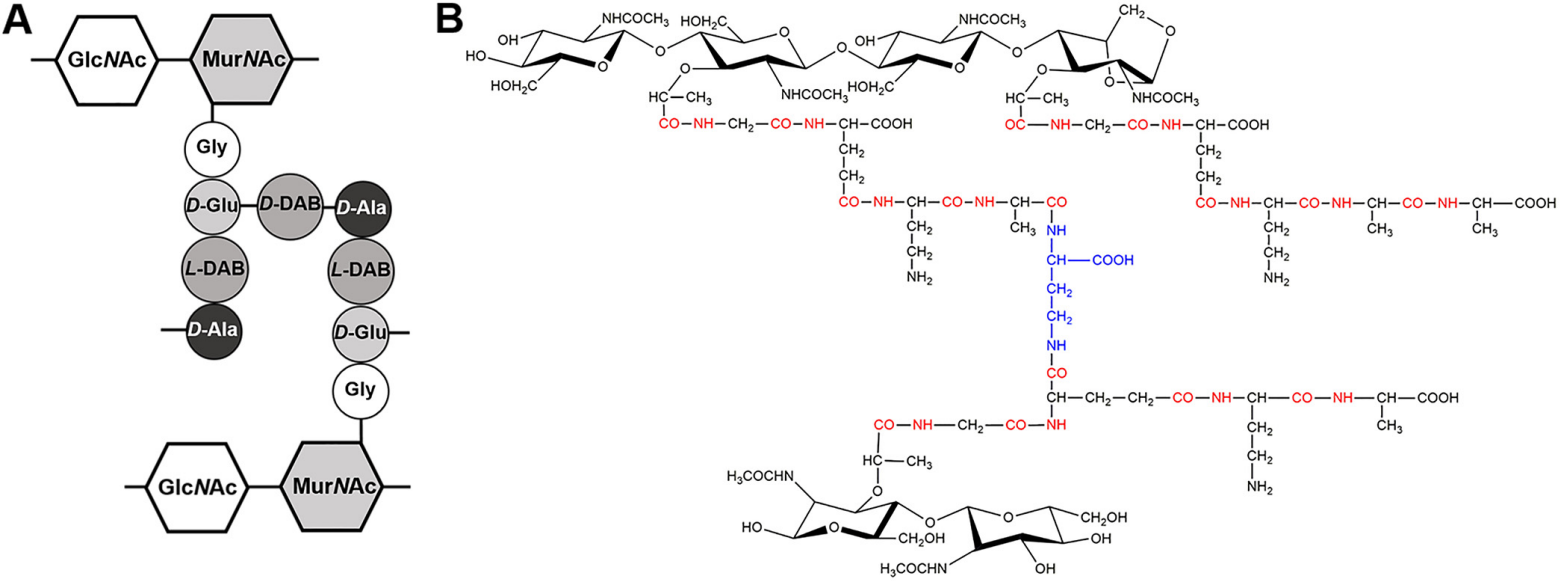
Structure of peptidoglycan of Clavibacter michiganensis. (A) Schematic structure of muropeptides of C. michiganensis. The basic structure Pentapeptide consists of the disaccharide (Glc*N*Ac-Mur*N*Ac) with a peptide chain (Gly - *D-Glu* - *L*-DAB - *D-Ala*). *D-DAB* constitutes the interpeptide bridge, which form indirect cross-links between two adjacent peptide chains. The peptidoglycan structure was designed with reference to Sasaki et al. (6). (B) Chemical composition of peptidoglycan of C. michiganensis. The glycopeptide linkage and peptide linkage (-CO-NH-) are shown in red and the interpeptide bridge is presented in blue.

Peptidoglycan, the major structure of cell walls in Gram-positive bacteria, is responsible for cellular morphology maintenance and protecting cells from external damages (1, 2). The peptidoglycan is composed of a glycan backbone and short cross-linked peptide chains (3). The glycan strands are cascaded of *N-acetylglucosamine* (Glc*N*Ac) and *N*-acetylmuramic acid (Mur*N*Ac) linked via β-(1,4) bonds (4), while the peptide chains are attached to the muramyl moiety to provide a mesh-like structure and this structure exhibited diversity among different species (5). In Gram-positive bacterium Clavibacter michiganensis, the peptide chains consist of 4 amino acids in order of Gly, *D-Glu*, *L*-diaminobutyric acid (*L*-DAB) and *D-Ala*. Peptide side chains from different Mur*N*Ac are cross-linked by *D-DAB* between *D-Glu* and *D-Ala* (2→4), which is B2γ variation peptidoglycan based on the classification system of Schleifer & Kandler (Fig. 1) (1, 6).

The peptidoglycan structure changes during bacterial growth and environmental fluctuation (7). Although the peptidoglycan structure in most species of bacteria has been generally characterized, the peptidoglycan architecture and dynamics are still elusive. Several peptidoglycan architectural models, including layered model, scaffold model and coiled-coil model, have been proposed to account for the insertion of nascent peptidoglycan and dynamic balance during cell growth and division. These models differ in the arranged directions of glycan strands and peptide side chains (8–12). In the layered model, the glycan strands of peptidoglycan run in parallel with the plasma membrane, while the scaffold model proposes that the glycan strands are oriented perpendicular to plasma membrane (13, 14). However, the hypotheses of layered model and scaffold model are controversial based on the glycan strand lengths and atomic force microscopy (AFM) observation in Bacillus subtilis and Lactococcus lactis cells, and the coiled-coil model was proposed that the peptidoglycan cables run parallel to the short cell axis (8, 9, 15).

Biosynthesis of peptidoglycan begins in cytoplasm and comes to an end in extracytoplasmic domain, where polymerizing and cross-linking (16). Penicillin-binding proteins (PBPs), which initially identified and named as targets of β-lactam antibiotic penicillin, are membrane-bound enzymes and involved in the last stage of peptidoglycan synthesis by catalyzing the maturation of peptidoglycan into a mesh-like structure (17, 18). The PBPs can be divided into class A high-molecular weight (HMW) PBPs, class B HMW PBPs, and low molecular weight (LMW) PBPs. Class A HMW PBPs are bifunctional enzymes, containing a transglycosylase domain and a transpeptidase domain, class B HMW PBPs consist of a transpeptidase domain and a domain with unknown function, both of class A and class B HMW PBPs are essential for the cross-linking of peptidoglycan (19–21). Especially, the glycosyltransferase domain of class A HMW PBPs could polymerize lipid II monomers to peptidoglycan chain, and the transpeptidase domain cross-links the stem peptides strands. LMW PBPs, which harbor a carboxypeptidase domain, are responsible for the cleavage of the fifth *D-Ala* from peptide chains (22). The relative expression of several PBPs was enhanced in *Vibrio parahaemlyticus* and Enterococcus faecalis under the viable but nonculturable (VBNC) state, and accordingly the thickness of cell wall or the cross-linkage of peptidoglycan was also increased (23–25). The VBNC state is a common survival strategy for non-sporulating bacteria to cope with adverse environmental conditions (26–28). It has been reported that VBNC cells of Vibrio vulnificus are more resistant to heat and oxidative stress (29). Our previous study reported that VBNC cells of C. michiganensis were more tolerant to a wide variety of stresses than culturable cells, and the lack of class A HMW PBP (*pbpC*) significantly reduced the resistance of log phase cells to stresses (30). However, the functions of PBPC on peptidoglycan assembly and architecture, and the mechanism on stress response of VBNC C. michiganensis cells are still unclear.

Here, we investigated the cell wall mechanical resistance of wild type and *ΔpbpC* mutant in log phase and VBNC state of C. michiganensis. The high-resolution AFM analysis and ultra-performance liquid chromatography-tandem mass spectrometry (UPLC-MS) were combined to explore the changes of peptidoglycan architecture and peptidoglycan compositions under Cu^2+^ stress. The results revealed an unexpected peptidoglycan response pattern to stress in wild type and *ΔpbpC* strains, which expanded our understandings and provided new insights into resistance mechanism of pathogenic bacteria.

## RESULTS

### The effect of *pbpC* on cell wall mechanical resistance in response to Cu^2+^.

**FIG 2:**
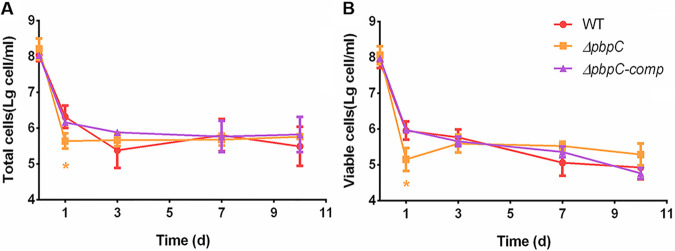
Survival curves of *ΔpbpC* when exposure to 50 μM Cu^2+^. Cells of Clavibacter michiganensis wild type (WT), *ΔpbpC* and *ΔpbpC-comp* were collected from solid medium for Cu^2+^ induction. The titers of total (A) and viable (B) cells were measured at 0 h, 1 d, 3 d, 7 d and 10 d by flow cytometry in conjunction with SYTO 9 and propidium iodide staining. All strains lost culturability after 1 day Cu^2+^ treatment and entered into the VBNC state. This experiment was performed three times, and error bars indicate standard deviation (SD). *, *P < *0.05.

It has been proved that loss of *pbpC* had significantly reduced survivorship of VBNC cells induced by Cu^2+^ and also increased the sensitivity to a series of stress treatments in C. michiganensis (30). To evaluate whether *pbpC* is required for stress resistance in VBNC state, the mutant *ΔpbpC* was induced into VBNC state, followed by ultrasonication treatment. Compared with wild type, *ΔpbpC* cells lost the culturability at 4 h when exposed to 50 μM Cu^2+^ (Fig. S1), and the most prominent exception in cell counts was that the titers of total and viable cells of *ΔpbpC* decreased significantly at 1 d post-treatment (Fig. 2). However, *ΔpbpC* had no significant difference at 3 d, 7 d, and 10 d post-treatment. As expected, there was no significant difference in total cells and viable cells between wild type and the complementation strain *ΔpbpC-comp*.

**FIG 3:**
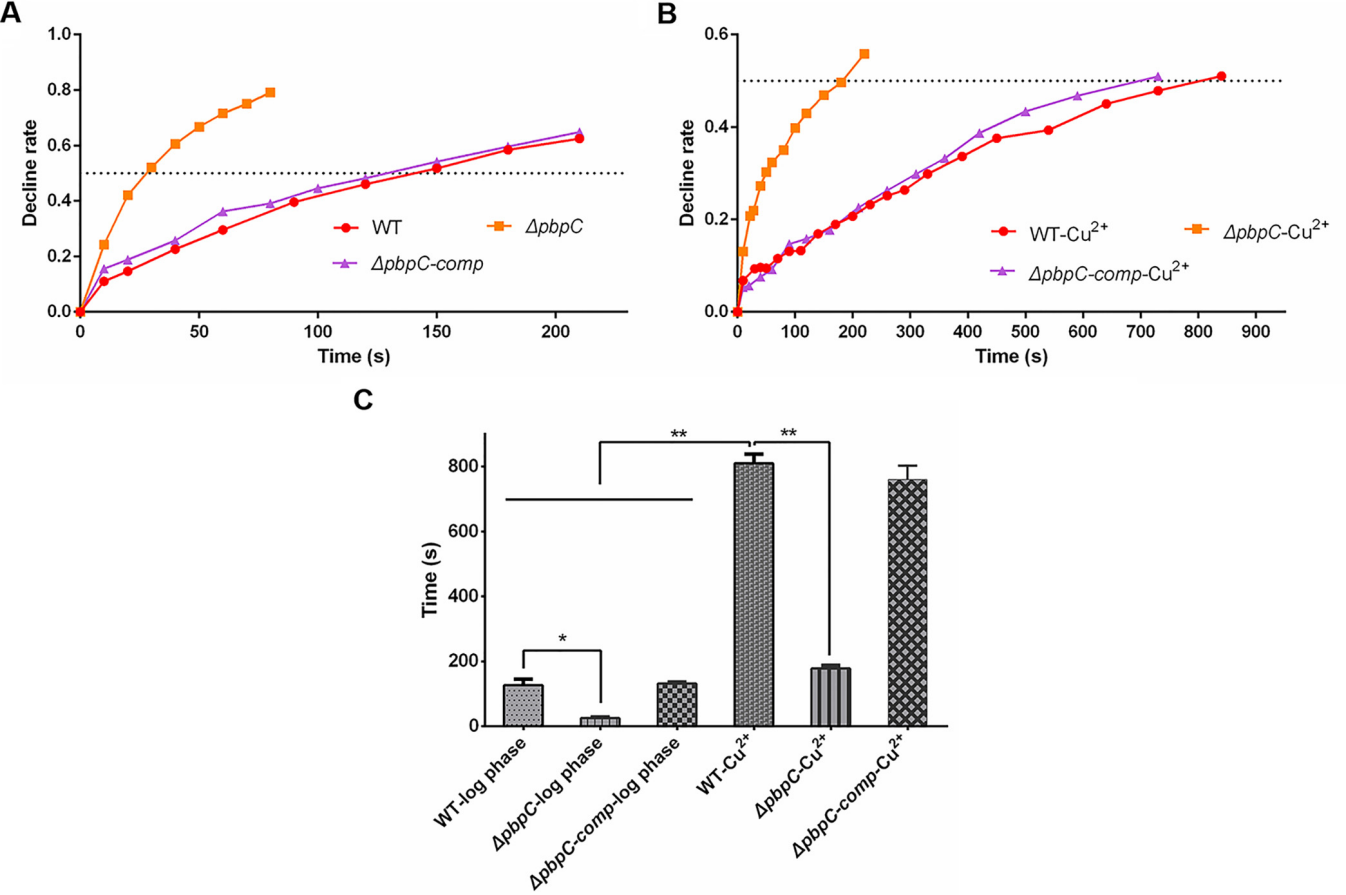
Mechanical resistance to ultrasonication of Clavibacter michiganensis cells. C. michiganensis wild type (WT), *ΔpbpC* and *ΔpbpC-comp* in log phase (A) were collected from LB solid medium and resuspended in 0.85% (wt/vol) NaCl because of the low amount of *ΔpbpC* in liquid medium. The OD_580_ was measured during ultrasonication process. The decline rate of cells in log phase (A) and VBNC state (B) was calculated and illustrated by dividing the initial OD_580_ with instant OD_580_ after every ultrasonication round. The duration time which 50% cells were decomposed was recorded to compare the mechanical resistance among different strains (C).

Both cells in log phase and 3 d post-treatment of Cu^2+^ were collected and adjusted to OD_580_ = 1.0 for mechanical resistance evaluation. The mechanical resistances of C. michiganensis cells were carried out by analysis of the changes of optical density during ultrasonication disruption. The decline rate of *ΔpbpC* in log phase was more rapidly than that of wild type, which was also observed in VBNC state (Fig. 3A and B). The times required for optical density to decrease to 50% were 178 s, 810 s and 760 s under sonication pulses treatment for VBNC cells of *ΔpbpC*, wild type and *ΔpbpC* complementation strains, compared to 25 s, 127 s and 133 s for that of log phase cells, respectively (Fig. 3C). The results revealed that the ability to resist external mechanical damage was greatly improved in VBNC cells, and the deficiency of PBPC led to a significant decrease in mechanical resistance in C. michiganensis cells in both log phase and VBNC state induced by Cu^2+^.

### The effect of *pbpC* on peptidoglycan architecture in response to Cu^2+^.

C. michiganensis cells lacking *pbpC* exhibited an apparent difference in cell morphology as observed by AFM. The *ΔpbpC* cells had a swollen appearance (Fig. S2C and D) and was not typical rod-shaped cells like wild type cells (Fig. S2A and B). Complementation of *pbpC* (*ΔpbpC-comp*) recovered the bacterial morphology similar to wild type (Fig. S2E and F). Further, aggregation of intracellular components was observed in both wild type and *ΔpbpC-comp* cells when exposure to Cu^2+^ for 3 d, as well as exosmose of intracellular material (Fig. S2G, H, K, and L). *ΔpbpC* cells exhibited aggravated morphology deformities under Cu^2+^ stress, possessing smaller coccus-like form and severe material aggregation in the intracellular and exudation of intracellular material (Fig. S2I and J). These results were consistent with the observation by scanning electron microscopy (30), and confirmed that the lack of *pbpC* reduced the bacterial resistance to Cu^2+^ in C. michiganensis.

**FIG 4:**
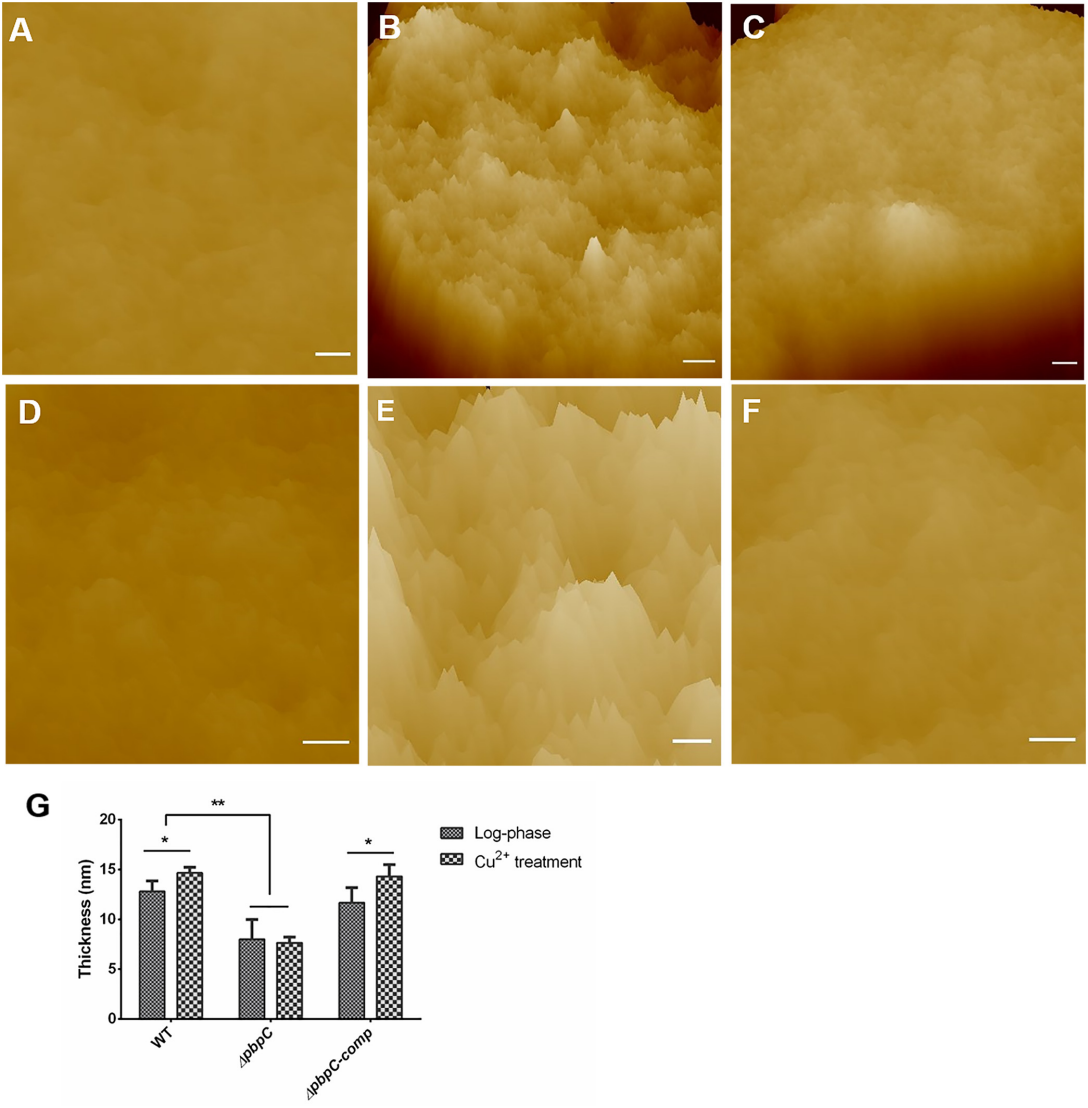
High-resolution atomic force microscopy images of peptidoglycan of Clavibacter michiganensis. The upper panels show the three-dimensional height images of peptidoglycan samples, which were extracted from C. michiganensis cells in log phase, including wild type (WT) (A), *ΔpbpC* (B) and *ΔpbpC-comp* (C). Images (D), (E), (F) were the three-dimensional height images of peptidoglycan extracted from wild type, *ΔpbpC* and *ΔpbpC-comp* under the treatment of 50 μM Cu^2+^ for 3 days respectively. Bar = 20 nm. (G), Thickness comparision of peptidoglycan layers from wild type WT, *ΔpbpC* and *ΔpbpC-comp* in log phase or under the treatment with Cu^2+^. Significant differences were detected with ANOVA followed by Tukey’s multiple-comparison test (*n* = 10, error bars indicate SD). *, *P < *0.05; **, *P < *0.01.

The thickness of the bacterial cell wall was determined to evaluate the role of *pbpC* on peptidoglycan architecture in response to Cu^2+^ stress. The peptidoglycan sacculi of wild type, *ΔpbpC* and *ΔpbpC-comp* were isolated and purified respectively, and then visualized by AFM. The average cell wall thickness of wild type and *ΔpbpC-comp* were 12.83 ± 0.85 nm and 11.67 ± 1.25 nm in log phase, and increased to 14.67 ± 0.47 nm and 14.33 ± 0.94 nm after exposure to Cu^2+^ stress for 3 d, respectively. However, the cell wall thickness of *ΔpbpC* was always around 8 nm with or without Cu^2+^ treatment (Fig. 4G), which indicated that lack of *pbpC* affected the bacterial cell wall synthesis and thickness in log phase, and impaired the ability of responding to Cu^2+^ stress by thickening the cell wall.

Purified sacculi of C. michiganensis were observed by high-resolution AFM. Exposed inner surface and outer surface of peptidoglycan of wild type in log phase were imaged, and no obvious or regular difference was observed between them (Fig. S3). A relatively rough surface feature of inner surface was not observed in C. michiganensis, which was different from the characteristics of B. subtilis strains (8, 31). The architecture of purified peptidoglycan of wild type, *ΔpbpC*, *ΔpbpC-comp* in different states were imaged. In log phase, peptidoglycan of wild type showed an irregular structure, but the reported “cable-like” resembled model of peptidoglycan (8) was not observed (Fig. 4A). The peptidoglycan layer of C. michiganensis was dense and smooth, which might be a network like structure (Fig. S3, Fig. 4A). Though the cell wall thickness of wild type increased significantly when exposed to Cu^2+^, there was no difference in density or architecture features in peptidoglycan layer (Fig. 4A, D, and G). However, a structural difference was observed in peptidoglycan of *ΔpbpC* compared to that of wild type. Peptidoglycan of *ΔpbpC* in log phase seemed to be sparser than that of wild type and featured with “ridge-and-groove” appearance (Fig. 4B). This “ridge-and-groove” character was more noticeable after Cu^2+^ treatment (Fig. 4E). However, the size of these “ridges” was irregular, large, or small (Fig. 4B and E). The appearances and thickness of peptidoglycan layer were restored after the complementation of *pbpC*, and the *ΔpbpC-comp* exhibited similar cell wall characteristics with wild type before and after Cu^2+^ treatment (Fig. 4C, F, and G).

### Peptidoglycan chemical composition characterization by UPLC-MS in response to Cu^2+^.

The UPLC-MS method was adopted to determine the structure of peptidoglycan for C. michiganensis wild type, *ΔpbpC*, and *ΔpbpC-comp* in log phase and during the treatment with Cu^2+^. The muropeptides were extracted and digested, then the digested fractions were collected from 2 to 30 min and each fraction was analyzed by ESI-TOF MS in positive ion mode (Fig. S4). Based on mature peptidoglycan composition structure of C. michiganensis (Fig. 1) (6), the structure of muropeptides was detected by UPLC-MS and analyzed. Finally, a total of 53 chemically different muropeptides were detected and classified, including 10 monomers, 20 dimers, 21 trimers and 2 tetramers. The monomeric peaks started from 2.67 to 17.78 min of retention time and the structures were displayed in Table S1. Pentapeptide which is the basic structure of muropeptides, was verified during UPLC-MS. The total ion current (TIC) chromatograms obtained by UPLC-MS of C. michiganensis wild type, *ΔpbpC* and *ΔpbpC-comp* in log phase and under Cu^2+^ stress treatment were illustrated in Fig. S4, and the complete results of muropeptides were shown in Table S1 for more details. The peptidoglycan mapping of these strains showed the same trend. A number of modifications, including the amidation or hydroxylation of *d-Glu*, the acetylation of Mur*N*Ac or *L*-DAB, the Mur*N*Ac modification to 1,6-anhydro Mur*N*Ac due to NaBH_4_ reduction reaction, and the loss of a Glc*N*Ac moiety, were identified on muropeptides in ESI mode (Table S1).

**FIG 5:**
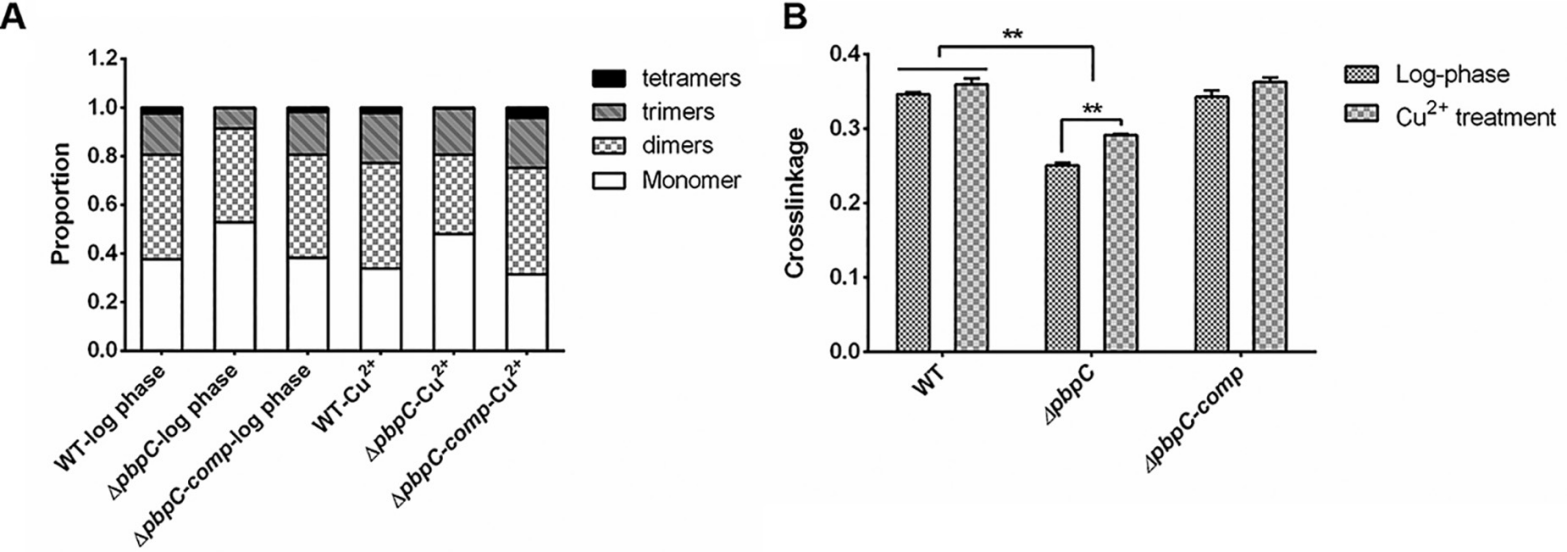
Deletion of *pbpC* influenced the response of cell wall peptidoglycan to Cu^2+^ treatment in Clavibacter michiganensis. (A) Distribution of muropeptides in wild type (WT), *ΔpbpC* and *ΔpbpC-comp* in different states. (B) Crosslinkage of peptidoglycan of wild type (WT), *ΔpbpC* and *ΔpbpC-comp* in different states. Significant differences were detected with ANOVA followed by Tukey’s multiple-comparison test (*n* = 2, error bars indicate SD). *, *P < *0.05; **, *P < *0.01.

Based on the muropeptides composition analysis, the muropeptide distribution and quantification of peptidoglycan in PBPC deficiency mutant and wild type were revealed. Compared to the wild type (37.59% monomer, 43.00% dimers, 17.07% trimers and 2.34% tetramers), the muropeptide profile of *ΔpbpC-comp* showed that the proportion of monomers of *ΔpbpC* mutant were increased to 52.77% which was offset by an equivalent reduction in the dimers (38.55%), trimers (8.43%) and tetramers (0.25%) in log phase (Fig. 5A). After Cu^2+^ treatment, the peptidoglycan of wild type contained fewer monomers, and more trimers and tetramers. The *ΔpbpC-comp* exhibited similar tendency as the wild type. Differently, after Cu^2+^ treatment, the proportions of monomers and dimers in *ΔpbpC* mutant decreased to 47.99% and 32.62% respectively, while the trimers and tetramers increased to 19.09% and 0.31% (Fig. 5A). In this way, the cross-linkage of muropeptides in wild type slightly increased from 34.64% in log phase to 35.97% after Cu^2+^ treatment for 3 days. The same trend was also found in *ΔpbpC-comp*. It was worth noting that the cross-linkage of peptidoglycan of *ΔpbpC* increased significantly by 4.1% after Cu^2+^ treatment compared with that of log phase, from 25.08% to 29.18%, but still lower than that of wild type and *ΔpbpC-comp* (Fig. 5B). These results suggested that the cell wall could not be thickened due to the lack of peptidoglycan synthase PBPC, the *ΔpbpC* mutant had to respond to Cu^2+^ stress by increasing the cross-linking of residual muropeptides.

### Evaluation of exoenzyme and exopolysaccharides of *ΔpbpC* cells.

**FIG 6:**
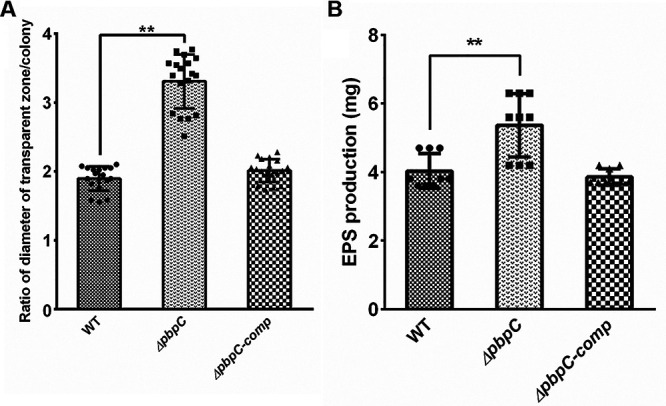
Loss of *pbpC* affected the production of amylase and sugar components in Clavibacter michiganensis. (A) Loss of *pbpC* intensified the secretion of amylase, which may be due to the sparse and lower cross-linkage of cell wall peptidoglycan in *ΔpbpC* mutant. (B) The production of exopolysaccharides (EPS) in wild type (WT), *ΔpbpC*, *ΔpbpC-comp* were measured by ethanol precipitation method. Asterisks (**) indicate significant differences between WT and *ΔpbpC* as calculated by a two-sided one sample *t* test (*P < *0.01). Error bars indicate SD.

Given the effect of thinner and reduced cross-linkage of peptidoglycan from *ΔpbpC* on cellular substance exudation, the production of amylase and exopolysaccharides (EPS) in wild type, *ΔpbpC*, and *ΔpbpC-comp* were evaluated. As expected, significant differences (*P < *0.01) in the production of amylase and EPS were determined between wild type and *ΔpbpC* mutant. The ratio of diameter of transparent zones/colony generated by amylase of *ΔpbpC* was around 1.5 fold of that of wild type and *ΔpbpC-comp*, which indicated the enhanced amylase secretion in *ΔpbpC* mutant (Fig. 6A). Additionally, the accumulation of more EPS was also measured in *ΔpbpC* mutant. As expected, no significant difference was found between complementation strain *ΔpbpC-comp* and wild type (Fig. 6B).

## DISSCUSION

Peptidoglycan, the main component of bacterial cell walls, is essential for viability and cell shape determination (32). It has a complex and dynamic structure for continuous cell growth and response to environmental stimuli (33). Three models for basic peptidoglycan architecture of Gram-positive bacteria have been proposed, and coiled-coil model has been reported in B. subtilis and L. lactis sacculi, in which glycan strands coil tightly to a peptidoglycan “rope,” then wrap around cells circumferentially (8, 9, 31). However, 3D visualization by electron cryotomography showed that the peptidoglycan of B. subtilis is a uniformly dense fabric, which conflicts with the coiled-coil model (15). Our work confirmed that the regular structure of peptidoglycan, such as “cable-like” structure did not exist in C. michiganensis. The peptidoglycan of C. michiganensis was dense and smooth without any noticeable feature to form a network like structure. The cell wall thickness was about 13 nm, which was thicker than that of Gram-negative bacterium Pseudomonas aeruginosa (3 nm) and Gram-positive bacterium B. subtilis (9 nm) (8, 34). This could be the reason for high tolerance of C. michiganensis cells to lysozyme and high sensitivity to β-lactam antibiotics. These results also indicated the diversity in peptidoglycan architecture for different species.

**FIG 7:**
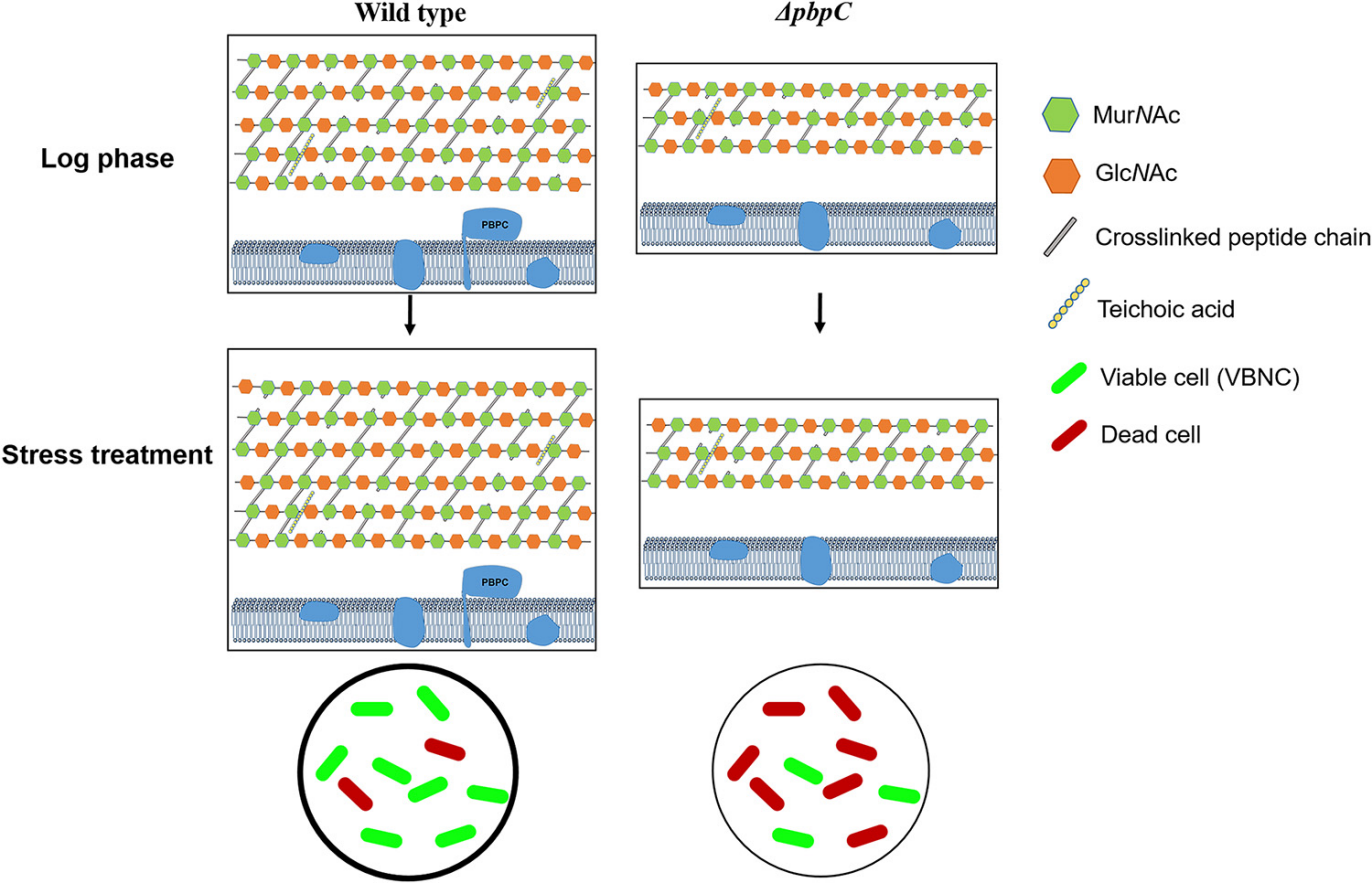
Model depicting the peptidoglycan stress response strategy in Clavibacter michiganensis. Lack of *pbpC* decreased the thickness of peptidoglycan layer and cross-linking degree in log phase cells, and changed the peptidoglycan response strategy to Cu^2+^ stress. The lower cross-linkage of peptidoglycan and thinner cell wall layer leads to the instability of VBNC state and more bacteria cell death.

Although the VBNC state was considered a survival strategy that protects bacteria from unsuitable conditions, there was no report about the relationship between the VBNC state and peptidoglycan changes in Gram-positive rod-shaped bacteria. The cross-linkage of peptidoglycan from VBNC enterococcal cells increased by 8%, and the VBNC cells were more resistant to mechanical disruption (25). Our results showed that when C. michiganensis cells were exposed to copper, there were a significant increase in cell wall thickness and a slight increase in cross-linkage of peptidoglycan, but different architecture of cell wall peptidoglycan was not observed by AFM imaging (Fig. 4). The VBNC cells induced by Cu^2+^ were more resistant to ultrasonication treatment than cells in log phase. It has been shown that peptidoglycan assembly relies on PBPs biosynthetic machinery (35). What is more, previous PBP evaluation in C. michiganensis has clearly indicated that the bifunctional enzyme, PBPC, played an important role in abiotic stresses resistance (30). Given the similar structure between the *d-Ala* - *d-Ala* and β-lactam antibiotic, the mutant *ΔpbpC* also exhibited great sensitivity to β-lactam antibiotic cefotaxime (Fig. S5), which was in accordance with the results of increased sensitivity to β-lactam antibiotic in *pbps* mutants of Staphylococcus aureus and Corynebacterium glutamicum (22, 36). It needs to be emphasized that differences in sensitivity to β-lactam antibiotic of the mutant was due to a decrease in the number of PBP targets, which also indicated the changes of peptidoglycan architecture due to lack of PBPC. Our study also revealed that the assembly of peptidoglycan and the complicated architecture of C. michiganensis cells changed under the control of PBPC. In log phase, the thickness of peptidoglycan layer and cross-links decreased with the loss of *pbpC*, this variation might be related to the architecture changes of peptidoglycan. Moreover, when exposed to copper, the *ΔpbpC* cells exhibited significant increased cross-linkage of muropeptides without the thickening of peptidoglycan, which resulted in mostly dead cells (30). Furthermore, the thickness of peptidoglycan in wild type cells increased, not accompanying with a significant increase in cross-link after treatment with copper (Fig. 7). The higher cross-linkage will create a more robust cell wall, and the thickness of peptidoglycan may play a more significant role in stress resistance. It was speculated that the synthesis of peptidoglycan was blocked in *ΔpbpC* mutant, then the cross-link machinery was enhanced to strengthen the resistance to adversity, which could explain the increase of resistance to mechanical disruption in VBNC *ΔpbpC* cells. Based on the PBPs analysis in C. michiganensis, the cross-link machinery might also be supported by the additional high-molecular-weight PBPs with putative transpeptidase activity, such as PBPA and PBPB1 (30). However, the attempts to delete PBPA and PBPB1 were unsuccessful, it would be more meaningful to obtain the available inducible promoter to regulate the expressions of PBPA and PBPB1 in C. michiganensis for function analysis. Further studies on exploring the relationship between transpeptidase activity and peptidoglycan response to stresses will provide essential clues to explain the dynamic changes of bacterial cell walls.

The increase of muropeptide monomers and the decrease of dimers, trimers, and tetramers were confirmed to be caused by lack of *pbpC*, which also give rise to a sparser peptidoglycan architecture and secreting more amylase and EPS. The more secretion components could account for the enhanced pathogenicity of *ΔpbpC* cells (30). Crosslink of peptide chains in peptidoglycan is an essential and complex process (37). The elongation and division of bacterial cells are achieved by cross-linking and polymerization of nascent peptidoglycan (19, 38). There are differences in the cell wall growth mode of rod-shaped bacteria. In E. coli and B. subtilis, the bacterial cells elongate by adding nascent peptidoglycan at dispersed sites throughout the cell cylinder, but by insertion of new peptidoglycan into the cell poles in Corynebacterineae suborder (16, 39). Given the morphology and muropeptide composition changes of C. michiganensis cells in response to stresses in the absence of PBPC, the insertion of new peptidoglycan by other PBPs in C. michiganensis may be affected. In *ΔpbpC* cells of C. michiganensis, it can be summarized that the cell wall polar growth remains normal, but the lateral insertion is disordered, which resulted in the swollen appearance and non-rod-shaped. In a word, the current study indicated that the exact insertion of new peptidoglycan in C. michiganensis was still not clear, but the class A HMW PBP PBPC is responsible for the lateral cell wall insertion for rod-shaped maintenance.

Overall, our study reinforces the importance of peptidoglycan in bacterial adaptation to environmental changes. The peptidoglycan assembly protein PBPC mediates the thickening of the cell wall and enhanced cross-link of peptidoglycan peptide chains in C. michiganensis when exposed to Cu^2+^ stress. The stress response strategy by significantly increasing peptidoglycan cross-linkage in *ΔpbpC* mutant elucidates the role of class A HMW PBP in Gram-positive rod-shaped plant pathogenic bacteria and provides new insights into the stress resistance mechanism in cell wall peptidoglycan development, which would give a great referential value to bacterial survival and physiology.

## MATERIALS AND METHODS

### Bacterial strains and culture conditions.

The bacteria strains C. michiganensis wild type, mutant *ΔpbpC*, complement *ΔpbpC-comp* used in this study were obtained from our previous report (30). All C. michiganensis strains were grown at 28°C on LB (10 g/l tryptone, 5 g/l yeast extract, 5 g/l NaCl) agar for 72 h or in LB broth for 20 h. Because the *ΔpbpC* mutant could not grow well in LB broth, the cells of wild type, *ΔpbpC* and *ΔpbpC-comp* in log phase for subsequent experiments were collected by picking colonies from LB agar.

The VBNC cells of C. michiganensis were induced at the initial concentration of 10^8^ CFU/mL by 50 μM Cu^2+^ via the method described in previous studies (30, 40). The number of viable cells was quantified by flow cytometry (FCM) at 0 d, 1 d, 3 d, 7 d and 10 d after exposure to Cu^2+^, meanwhile the titer of VBNC cells was calculated by subtracting the number of culturable cells from viable cells.

### Evaluation of cell wall resistance.

The cell wall resistance of VBNC C. michiganensis cells induced by Cu^2+^ was assessed by ultrasonication treatment. The pellets of wild type, *ΔpbpC* and *ΔpbpC-comp* in log phase or VBNC state were resuspended in 15 mL 0.85% (wt/vol) NaCl and the final titer was adjusted to OD_580_ of 1.0. Then, ultrasonication at 50% power was applied to bacteria aliquots, along with the concentration measurement at regular intervals (6 s) until the suspension was clear.

### Observation of *C. michiganensis* saccule by AFM.

Cells in log phase or VBNC state were harvested, then the corresponding peptidoglycan was purified as previous description (8, 31). Briefly, collected cells were washed, and boiled for 10 min, then broken by French press (Constant Systems). Broken sacculi were concentrated and boiled in 5% SDS for 1 h. The extract after treatment was washed for three times until no SDS residue. Finally, the samples were incubated in 48% hydrofluoric acid (HF) at 4°C for 24 h to remove accessory polymers, including teichoic acid, then washed for three times. For AFM imaging, purified sacculi were diluted in MilliQ water and air dried onto mica.

AFM images were recorded by using Multimode VIII AFM with Nanoscope V controller (Bruker AXS) with an OMV optical microscope in scanasyst mode. Sharpened silicon cantilevers (XSC11/AL BS, MikroMash) were used in ambient conditions.

### Muropeptides preparation for UPLC-MS.

The muropeptides used for UPLC-MS analysis were extracted and purified by SDS method (41–43). One milliliter of cells (OD_580_ = 20) in log phase or VBNC state were collected for muropeptides preparation. Cultures were boiled in 0.25% SDS solution for 30 min, then washed, and treated by sonifier water bath for 30 min. The attached components, including sugars and proteins, were removed by incubating with DNase (15 μg/mL), RNase (60 μg/mL), and trypsin (50 μg/mL) solutions at 37°C. To release teichoic acid, the pellet was incubated in 1 M HCl for 4 h at 37°C, 150 rpm. Afterwards, purified samples were resuspended in 12.5 mM sodium dihydrogen-phosphate buffer and digested with mutanolysin (M9901, Sigma-Aldrich) for 16 h, resulting hydrolysate was treated by reduction solution sodium borohydride (NaBH_4_) for 20 min, followed by adding 0.5 M formic acid to stop the reduced reaction. Finally, the prepared muropeptides could be analyzed by ultraperformance liquid chomatography mass spectrometry (UPLC-MS).

### UPLC-MS analysis.

The peptidoglycan mixtures were separated by UPLC using a Acquity CSH C18 column (1.7 μm, 2.1 by 100 mm) coupled to a Xevo G2 Q-TOF (Q-TOF G6520, Waters). The injection volume was 4 μL and flow rate was 0.2 mL/min. Buffer A was water containing 0.4% (v:v) formic acid, and buffer B was acetonitrile. A linear gradient from 2% to 15% buffer B over a period of 30 min was started after 10 min running with 2% buffer B. The MS was set to positive ESI mode with a scan range from 50–1600 *m/z*. The structures of muropeptides were analyzed based on the chemical composition of C. michiganensis peptidoglycan by MassLynx (1, 6) and illustrated with ChemDrawUltra 13.0.

### Determination of production of amylase and EPS.

The production of amylase of C. michiganensis wild type, *ΔpbpC* and *ΔpbpC-comp* were measured based on starch hydrolysis activity as previously reported (44) with a few modifications. Two microliter of overnight cultures (OD_580_ = 0.3) was inoculated on mM9 (6 g/l Na_2_HPO_4_•12H_2_O, 3 g/l KH_2_PO_4_, 1 g/l NH_4_Cl, 0.5 g/l NaCl, 1 mM MgSO_4_•7H_2_O, 0.01 mM CaCl_2_•2H_2_O, 2 g/l glucose, 200 mg/l methionine, 200 mg/l thiamin, 20 mg/l nicotinic acid) agar supplemented with 1.5% soluble starch (Sigma-Aldrich, CA). Incubate the colonies on the plates with Lugol’s iodine solution for 15 min. Afterwards, wash the plates with water and 70% ethanol. Then, the diameters of transparent zones were generated, and growing colonies were measured under purple background. The ratios of diameter of transparent zone/colony of wild type, *ΔpbpC* and *ΔpbpC-comp* were calculated to compare the amylase activity.

The determination of EPS was carried out by ethanol precipitation method (45). Sorbitol at final concentration of 0.1 M and 4% glucose were added in LB broth for shaking over 24 h. The supernatant of 10 mL bacterial suspension (OD_580_ = 1.0) was mixed with absolute ethanol in a 1: 2 volume ratio and incubated at −20°C overnight. Finally, the pellet was collected and dried in vacuum before weighing.
