# Supplementary material for: Class A Penicillin-Binding Protein C Is Responsible for Stress Response by Regulation of Peptidoglycan Assembly in Clavibacter michiganensis
Source: Microbiol Spectr. 2022 Aug 30;10(5):e01816-22. doi: 10.1128/spectrum.01816-22 (PMC9603630; doi:10.1128/spectrum.01816-22)
Supplement: Supplemental file 1 — Table S1 and Fig. S1 to S5. Download spectrum.01816-22-s0001.pdf, PDF file, 0.7 MB [file spectrum.01816-22-s0001.pdf]

SUPPLEMENTAL MATERIAL

Table S1 Muropeptides of *C. michiganensis* analyzed by UPLC-MS

Figure S1 Diagram of culturability test for *ΔpbpC* cells. The *ΔpbpC* cells from 10 ml bacterial suspension after exposure to 50 μM Cu^{2+} for 4 h were collected and placed on LB agar plate. No colony was grown after cultured for 72 h at 28°C, indicating the cells were in VBNC state (⩽1 CFU/mL).

Figure S2 Cell morphology of *ΔpbpC* in log phase or under Cu^{2+} stress treatment in *Clavibacter michiganensis*. The upper panels show the morphology of *C. michiganensis* cells in log phase observed by AFM, including wild type (A, B), *ΔpbpC* (C, D) and *ΔpbpC-comp* (E, F). The lower images show the morphology of cells when exposure to 50 μM Cu^{2+} for 3 days, including wild type (G, H), *ΔpbpC* (I, J) and *ΔpbpC-comp* (K, L). (A), (C), (E), (G), (I), (K), height images; (B), (D), (F), (H), (J), (L), peak force error images. Bar = 500 nm.

Figure S3 Atomic force microscopy images of *Clavibacter michiganensis* sacculi. The inner surface of *C. michiganensis* peptidoglycan was exposed in sacculi. (A) and (B), Peak force error image and height image of saccule of *C. michiganensis* wild type in log phase respectively. The corresponding enlarged three-dimensional height images of inner surface and outer surface was also illustrated in (C) and (D) respectively. (A), (B), bar = 500 nm, (C), (D), bar = 100

nm.

Figure S4 Deletion of *pbpC* affected the peptidoglycan patterns in *Clavibacter michiganensis*. (A), (B), (C), Muropeptide profiles of *C. michiganensis* wild type, *ΔpbpC* and *ΔpbpC-comp* in log-phase obtained by UPLC-MS. (D), (E), (F), TIC chromatograms of UPLC-MS analysis of wild type, *ΔpbpC* and *ΔpbpC-comp* when exposure to 50 μM Cu^{2+} for 3 days.

Figure S5 Deletion of *pbpC* affected the sensitivity of *Clavibacter michiganensis* cells to cefotaxime. Bacterial suspension of wild type cells, its deletion derivatives *ΔpbpC* and complementation strain *ΔpbpC-comp* were normalized to an OD_{580} of 0.3, serially diluted, and spotted 5 μl onto LB agar supplement with 0.25 μg/ml cefotaxime. Plates were incubated for 72 h at 28°C. Loss of *pbpC* increased the sensitivity for β-lactam antibiotic cefotaxime.

Table S1 Muropeptides of *C. michiganensis* analyzed by UPLC-MS

$M+H^+$ Measured	Proposed sum formula	Muropeptides	1,6-anhydro MurNAc	-GlcNAc	Amidation of Glu	Glu hydroxylation	Acetylation	Phosphate moiety bound
536.2089	C21H34N3O13	Di	1					
721.3181	C28H48N89O14	Penta		1	1			
783.3266	C30H50N6O18	Tri						
811.3229	C29H48N8O14	Tetra+DAB	1	1				
906.3861	C36H59N9O18	Penta			1		1	
996.4341	C39H65N9O21	Tetra+DAB					1	
1006.4713	C40H67N11O19	Penta(+DAB)	1		1			
1053.4570	C41H68N10O22	Tetra+DAB			1	1	2	
1067.4719	C42H70N10O22	Penta(+DAB)					1	
1127.4784	C44H74N10O24	Penta		+[a]	1			
1549.6862	C62H100N16O30	Penta-Tetra(+DAB)	1	2			2	
1661.8009	C67H108N18O31	Penta-Tetra(+DAB)	1	2	1		3	
1734.7602	C70H111N17O34	Tetra-Tetra(+DAB)	2	1			2	
1742.7374	C69H111N15O37	Tetra-Tri	1				1	
1791.7752	C67H111N18O37	Penta-Tetra		1	2	1	1	1
1809.7858	C72H116N18O36	Penta-Penta	1	1	1	1	2	
1805.7940	C70H116N16O39	Tetra-Tetra				1		
1813.6550	C72H116N16O38	Tetra-Tetra	1				1	
1822.6984	C68H108N15O41P	Penta-Di	1		1	2	1	1
1823.8054	C73H118N18O36	Penta-Tetra	2		2			
1840.7270	C68H110N15O42P	Penta-Di			1	2	1	1

1893.6320	C69H114N20O38P2	Penta-Tetra(+DAB)	1	1	2			2
1955.8390	C78H126N18O40	Penta-Tetra(+DAB)	1				2	
1973.8548	C78H128N18O41	Tetra-Tetra(+DAB)					2	
2012.8650	C80H129N19O41	Penta-Penta	1		1	1	2	
2026.8834	C81H131N19O41	Penta-Tetra(+DAB)	1				2	
2030.8806	C80H131N19O42	Penta-Penta			1	1	2	
2044.8964	C81H133N19O42	Penta-Tetra(+DAB)					2	
2083.7962	C78H131N20O44P	Tetra-Tetra(+DAB+DAB)				1		
2097.7938	C77H126N20O44P2	Penta-Tetra(+DAB)	1		1			2
2363.0438	C95H155N27O43	(Tetra)3(+DAB+DAB)	2	3	1		2	
2517.0720	C99H161N25O51	Penta-Tetra-Tri	2	1	1	2		
2584.1326	C103H170N28O49	(Tetra)3(+DAB+DAB)	1	2	1		2	
2684.1256	C107H170N26O54	Penta-Tetra-Tri	3		2	2		
2702.1499	C107H172N26O55	Penta-Tetra-Tri	2		1	2		
2738.1644	C107H176N26O57	(Tetra)3			1	2		
2751.2092	C111H179N29O52	(Tetra)3(+DAB+DAB)	3	1	1		2	
2765.2042	C112H177N27O54	Penta-(Tetra)2(+DAB)	3	1			3	
2769.1832	C111H181N29O53	(Tetra)3(+DAB+DAB)	2	1	1		2	
2787.2202	C111H183N29O54	(Tetra)3(+DAB+DAB)	1	1	1		2	
2791.0730	C104H169N25O60P2	(Tetra)2-Tri	1		1			2
2801.2268	C112H181N27O56	Penta-(Tetra)2(+DAB)	1	1			3	
2972.2963	C119H194N30O58	(Tetra)3(+DAB+DAB)			1		2	
2986.2922	C120H192N28O60	Penta-	2				3	

		(Tetra)2(+DAB)						
2987.2939	C119H195N31O58	(Tetra)3(+DAB+DAB)	2		2	1	2	
2990.2942	C119H196N30O59	(Tetra)3(+DAB+DAB)			1		2	
3004.2901	C120H194N28O61	Penta-(Tetra)2(+DAB)	1				3	
3008.3086	C119H198N30O60	(Tetra)3(+DAB+DAB)			1		2	
3022.3018	C120H196N28O62	Penta-(Tetra)2(+DAB)					3	
3061.1740	C115H195N31O62P2	(Tetra)3(+DAB+DAB)	1			2	1	2
3075.2266	C116H189N29O64P2	Penta-(Tetra)2(+DAB)	1		1		1	2
3476.4814	C136H219N36O68P	(Tetra)4		1	3	1	4	1
3715.5790	C144H236N37O75P	(Tetra)4			3	1	2	1

[a], the addition of Glc*N*Ac.

Figure S1

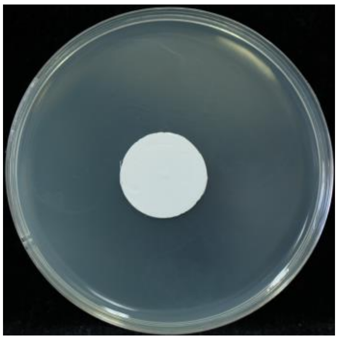

Figure S1 Diagram of culturability test for *ΔpbpC* cells. The *ΔpbpC* cells from 10 ml bacterial suspension after exposure to 50 μM Cu^{2+} for 4 h were collected and placed on LB agar plate. No colony was grown after cultured for 72 h at 28°C, indicating the cells were in VBNC state (⩽1 CFU/mL).

Figure S2

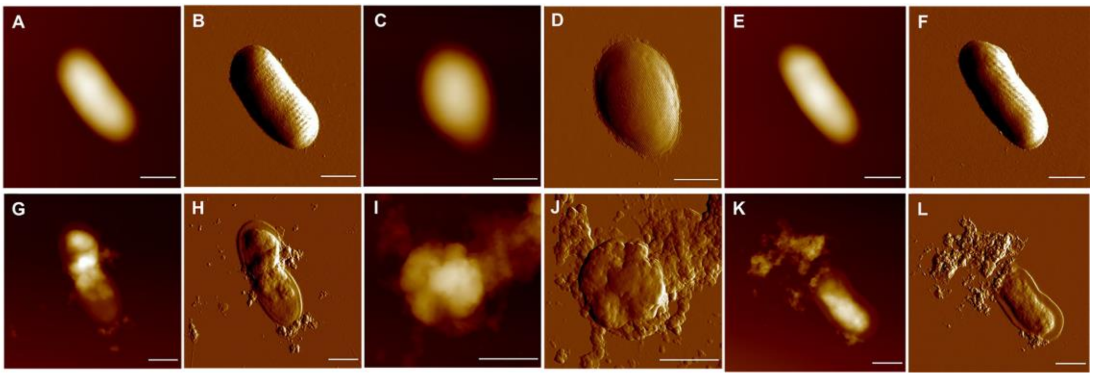

Figure S2 Cell morphology of *ΔpbpC* in log phase or under Cu^{2+} stress treatment in *Clavibacter michiganensis*. The upper panels show the morphology of *C. michiganensis* cells in log phase observed by AFM, including wild type (A, B), *ΔpbpC* (C, D) and *ΔpbpC-comp* (E, F). The lower images show the morphology of cells when exposure to 50 μM Cu^{2+} for 3 days, including wild type (G, H), *ΔpbpC* (I, J) and *ΔpbpC-comp* (K, L). (A), (C), (E), (G), (I), (K), height images; (B), (D), (F), (H), (J), (L), peak force error images. Bar = 500 nm.

Figure S3

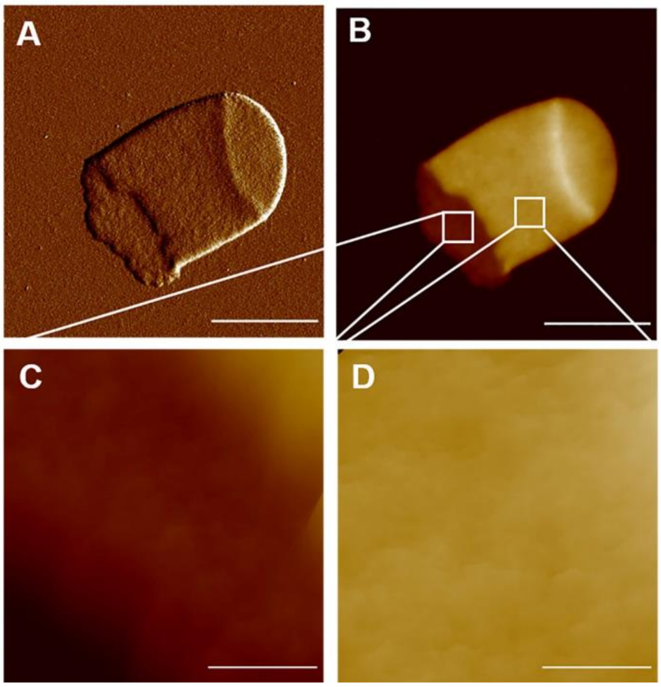

Figure S3 Atomic force microscopy images of *Clavibacter michiganensis* sacculi. The inner surface of *C. michiganensis* peptidoglycan was exposed in sacculi. (A) and (B), Peak force error image and height image of saccule of *C. michiganensis* wild type in log phase respectively. The corresponding enlarged three-dimensional height images of inner surface and outer surface was also illustrated in (C) and (D) respectively. (A), (B), bar = 500 nm, (C), (D), bar = 100 nm.

Figure S4

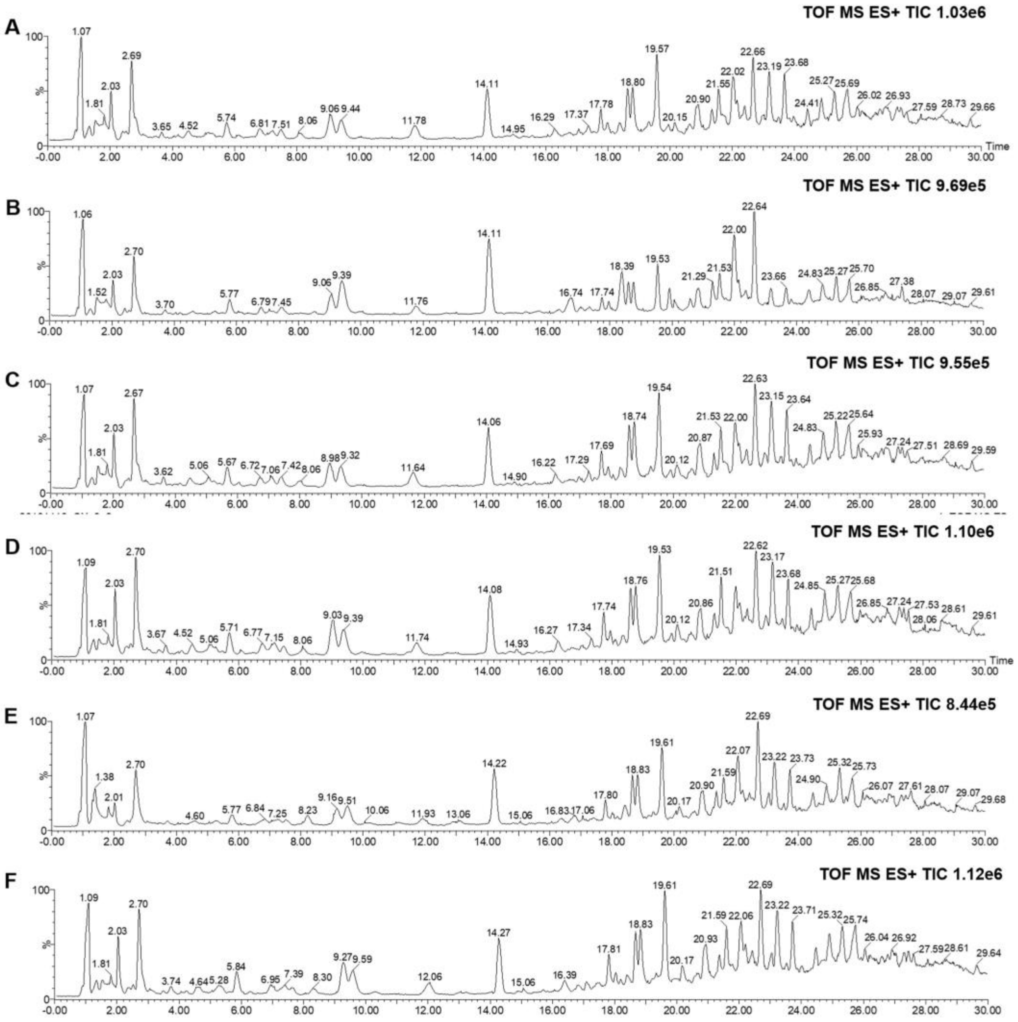

Figure S4 Deletion of *pbpC* affected the peptidoglycan patterns in *Clavibacter michiganensis*. (A), (B), (C), Muropeptide profiles of *C. michiganensis* wild type, *ΔpbpC* and *ΔpbpC-comp* in log-phase obtained by UPLC-MS. (D), (E), (F), TIC chromatograms of UPLC-MS analysis of wild type, *ΔpbpC* and *ΔpbpC-comp* when exposure to 50 μM Cu^{2+} for 3 days.

Figure S5

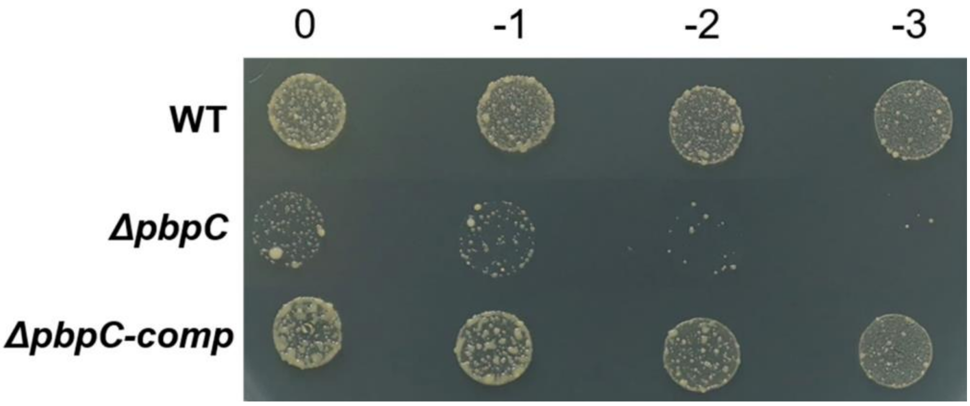

Figure S5 Deletion of *pbpC* affected the sensitivity of *Clavibacter michiganensis* cells to cefotaxime. Bacterial suspension of wild type cells, its deletion derivatives *ΔpbpC* and complementation strain *ΔpbpC-comp* were normalized to an OD_{580} of 0.3, serially diluted, and spotted 5 μl onto LB agar supplement with 0.25 μg/ml cefotaxime. Plates were incubated for 72 h at 28°C. Loss of *pbpC* increased the sensitivity for β-lactam antibiotic cefotaxime.
